# Dual-Mode Sensing Platform for Cancer Antigen 15-3 Determination Based on a Silica Nanochannel Array Using Electrochemiluminescence and Electrochemistry

**DOI:** 10.3390/bios13030317

**Published:** 2023-02-24

**Authors:** Jie Huang, Tongtong Zhang, Yanyan Zheng, Jiyang Liu

**Affiliations:** 1 Key Laboratory of Surface & Interface Science of Polymer Materials of Zhejiang Province, Department of Chemistry, Zhejiang Sci-Tech University, Hangzhou 310018, China; 2 Key Laboratory of Integrated Oncology and Intelligent Medicine of Zhejiang Province, Department of Hepatobiliary and Pancreatic Surgery, Affiliated Hangzhou First People’s Hospital, Zhejiang University School of Medicine, Hangzhou 310006, China

**Keywords:** dual-mode detection, electrochemiluminescence, electrochemistry, cancer antigen 15-3, vertically ordered mesoporous silica film, immunosensor

## Abstract

An electrochemiluminescence-electrochemistry (ECL-EC) dual-mode sensing platform based on a vertically-ordered mesoporous silica films (VMSF) modified electrode was designed here for the sensitive and selective determination of cancer antigen 15-3 (CA 15-3), a specific biomarker of breast cancer. VMSF was assembled through a rapid electrochemically assisted self-assembly (EASA) method and plays a crucial role in signal amplification via a strong electrostatic interaction with the positively charged bifunctional probe Ru(bpy_)3_^2+^. To construct the biorecognition interface, epoxy functional silane was linked to the surface of VMSF for further covalent immobilization of the antibody. As a benefit from the specific combination of antigen and antibody, a non-conductive immunocomplex layer was formed in the presence of CA 15-3, leading to the hinderance of the mass and electron transfer of the probes. Based on this strategy, the dual-mode determination of CA 15-3 ranging from 0.1 mU/mL to 100 mU/mL with a LOD of 9 μU/mL for ECL mode, and 10 mU/mL to 200 U/mL with a LOD of 5.4 mU/mL for EC mode, was achieved. The proposed immunosensor was successfully employed for the detection of CA 15-3 in human serum without tedious pretreatment.

## 1. Introduction

Cancer has become the second major cause of death worldwide [1]. According to the latest statistics from the International Agency for Research on Cancer (IARC), breast cancer occurs with the highest incidence and is also the major cause of cancer-related mortality in females [2], indicating that it is the most common and high-risk cancer globally. The diagnosis of breast cancer mainly relies on clinical screening or imaging analysis that often requires invasive means to obtain tissue samples and may bring sufferings to the patients [3]. The establishment of a noninvasive/minimally invasive, non-radioactive, simple, and rapid technique for early diagnosis remains a serious challenge and an unmet demand. Tumor markers, a kind of biological macromolecules existing in accessible body fluids (e.g., blood, serum, and urine) [4], provide a new standard for cancer diagnosis, health monitoring and personalized treatment. Cancer antigen 15-3 (CA 15-3) is an acidic glycoprotein with a molecular weight of 300–450 kDa that is often found in those who suffer from breast cancer [5,6]. The serum content of CA 15-3 in healthy people is usually less than 30 U/mL [7]. Abnormally elevated serum CA 15-3 levels indicate a higher risk of breast cancer. Simple but sensitive identification and quantitation of serum CA 15-3 level is critical for cancer screening, determination of disease progression, and treatment effect assessment.

Enzyme-linked immunosorbent assay (ELISA) [8], fluorescent immunoassay (FIA) [9], and radioimmunoassay (RIA) [10] have been reported for the quantitation of CA 15-3, but cumbersome instruments, trained operators and time-consuming preprocesses are always required. The electrochemical (EC) sensing technique has proven itself to be a powerful tool because of the fast response, simple operation, and ease of integration [11,12] compared with the traditional immunoassay, despite the hidden risk of inaccurate test results. To guarantee the accuracy and reliability of the diagnosis, dual-mode bioassays with different mechanisms and independent signal conversion have been developed. To date, a series of dual-mode biosensors which incorporate colorimetric-electrochemiluminescence (ECL) [13], ECL-electrochemical impedance spectroscopy (EIS) [14], fluorescence-colorimetry [15], and ECL-EC [16,17] have been reported. Among them, the ECL-EC dual-mode that combines the wider linear range and zero background signal of the former and the fast and easy operation characteristics of the latter with highly sensitivity, has attracted much attention. Generally, two different probes for ECL and EC are involved in the reported dual-mode detection strategy, which requires more laborious procedures. Tris(2,2′-bipyridyl) ruthenium (Ru(bpy)_3_^2+^), as an excellent bifunctional probe with both outstanding EC and ECL response, is a good candidate for constructing an ECL-EC dual-mode sensor, not only simplifying the manual work, but also reducing the analysis error effectively [17,18].

In the recent three decades, functional mesoporous materials possessing abundant unique mesostructures have exhibited tremendous potential in the fields of adsorption, separation, catalysis, and sensing [19,20,21]. Among them, the vertically-ordered mesoporous silica films (VMSF) made of numerous perpendicularly aligned nanochannels with a ultrasmall and uniform diameter of 2~3 nm has attracted much attention [22,23]. The deprotonation of plentiful silanols with p*K*_a_ of ~2 on the VMSF surface endows its negatively charged property [24,25]. All these characteristics endow VMSF with a good capability of prominent charge-based permselectivity, high molecular permeability, and outstanding antifouling ability [26,27,28]. Therefore, VMSF has been widely used in the construction of sensing platforms to serve certain functions (e.g., preconcentration through electrostatic interaction [29], hydrogen bonding [30] or hydrophobic extraction [31], specific recognition by tailoring recognitive molecules [32], and antifouling ability through size exclusion effect of nanopores [33]). As a monolayer of modification material with a nanoscale thickness, diverse VMSF-based sensors have been developed for the determination of biomolecules (DNA, antibodies, and antigens) [17,34], pharmaceutical molecules [35,36], small organic pollutants [30,37], and metal ions [38,39] in biofluids (blood, serum, urine, and sweat) and environmental samples. Su’s group observed a two-orders-of-magnitude ECL intensity enhancement when equipping indium tin oxide (ITO) electrode with a layer of VMSF due to the strong electrostatic attraction between the negatively charged nanochannels and the cationic ECL luminophore Ru(bpy)_3_^2+^ and obtained a high ECL intensity even with a very low concentration [40]. On the other hand, the existence of silanols with excellent covalent reactivity towards silane coupling reagents provides the precondition to construct a recognition interface by introducing certain specific molecules to design a gated sensing scheme [41,42]. VMSF has become a fascinating material in the construction of sensing interfaces, demonstrating a great potential for direct and sensitive detection of targets in complex samples without cumbersome pretreatment processes including filtration, separation, and pre-enrichment.

**Scheme 1:**
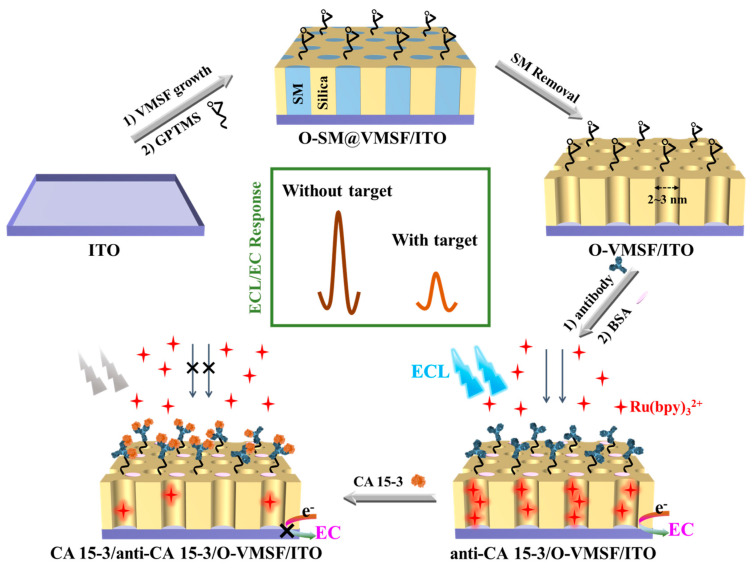
Illustration of the preparation of the immunosensor and detection of CA 15-3.

In this work, an ECL-EC dual-mode immunosensor constructed on an ITO substrate composed of a layer of mesoporous silica membrane as the signal amplifier, and a monomolecular layer of anti-CA 15-3 antibody as the recognition element was designed for the sensitive and selective detection of the breast cancer biomarker CA 15-3. Compared with medical imaging and pathological biopsy applied to the clinical diagnosis of breast cancer, this method offers more hope for the early detection of breast cancer rapidly and conveniently. As shown in Scheme 1, VMSF was first assembled on ITO through the electrochemical assisted self-assembly (EASA) method. The construction of the immune recognition interface for further identifying and capturing targets specifically was achieved by immobilizing the antibodies with the assistance of (3-glycidyloxypropyl)trimethoxysilane (GPTMS) linked to the outer surface of VMSF beforehand. A layer of immunocomplex with poor conductivity and large size would form in the presence of the CA 15-3 antigen and hamper the mass transfer and electron transfer of the bifunctional probe Ru(bpy)_3_^2+^, leading to decreased ECL-EC signal and the final signal-off detection of CA 15-3. The benefits of the immunosensor lie in the easy fabrication of the modification material which serves as both a signal amplifier to enrich Ru(bpy)_3_^2+^ and an anti-fouling filter, as well as in the generalizability of the detection strategy which is suitable for the quantitation of various targets by simply changing the immobilized bio-receptors.

## 2. Materials and Methods

### 2.1. Reagents and Solution

CA 15-3, anti-CA 15-3 antibody, carcinoembryonic antigen (CEA), and prostate specific antigen (PSA) were from Beijing KEY-BIO Biotech. Tetraethyl orthosilicate (TEOS), hexadecyl trimethyl ammonium bromide (CTAB), sodium nitrate (NaNO_3_), sodium hydroxide (NaOH), potassium ferrocyanide (K_4_[Fe(CN)_6_]), potassium ferricyanide (K_3_[Fe(CN)_6_]), bovine serum albumin (BSA), and (3-glycidoxypropyl)methyldiethoxysilane (GPTMS) were from Aladdin Chemistry Co. Ltd. (China). Tris(2,2-bipyridyl) dichlororuthenium (II) hexahydrate (Ru(bpy)_3_Cl_2_·6H_2_O) and hexaammineruthenium (III) chloride (Ru(NH_3_)_6_Cl_3_) were purchased from Sigma-Aldrich. Ethanol (99.8%), hydrochloric acid (HCl, 38%), and acetone were from Hangzhou Shuanglin Chemical Reagent Co. Ltd. Phosphate buffered saline (PBS, 0.01 M, pH = 7.4) was obtained by mixing NaH_2_PO_4_ and Na_2_HPO_4_ in a certain ratio. All chemicals and reagents were used as received without any further purification. All the aqueous solutions used here were prepared using ultrapure water (18.2 MΩ cm) from Milli-Q Systems (Millipore Inc., Massachusetts, America). Serum of healthy men was obtained from the Center of Disease Control and Prevention (Hangzhou, China) for real sample analysis.

### 2.2. Apparatus and Electrodes

An MPI-E II ECL analytical system (Xi’an Remex Analytical Instrument Ltd., Xi’an, China) was used to perform ECL measurements. EC measurements, including cyclic voltammetry (CV), differential pulse voltammetry (DPV), and electrochemical impedance spectroscopy (EIS) were performed on an Autolab electrochemical workstation (PGSTAT302N, Metrohm, Switzerland). A traditional three-electrode system was adopted. Bare or modified ITO, platinum wire or platinum plate, and Ag/AgCl electrode (saturated with KCl solution) were used as the working electrode, counter electrode and reference electrode, respectively. Zhuhai Kaivo Optoelectronic Technology provided ITO glasses (<17 Ω/square, thickness: 100 ± 20 nm), which were cleaned ultrasonically with 1 M NaOH, acetone, ethanol and deionized water, respectively. The geometric surface area of the electrode was fixed to 1 cm × 0.5 cm.

The morphology of VMSF/ITO was investigated on a JEM-2100 (JEOL Ltd., Tokyo, Japan) transmission electron microscopy (TEM). The thickness of VMSF/ITO was characterized with a SU8010 (Hitachi, Tokyo, Japan) scanning electron microscopy (SEM).

### 2.3. Procedures

#### 2.3.1. Preparation of VMSF/ITO Electrode

According to the reported electrochemically assisted self-assembly (EASA) method, an easy and rapid modification of VMSF on the ITO electrode was achieved [43]. A sol for film electrodeposition was prepared as follows: first mix 20 mL of ethanol and 20 mL of NaNO_3_ (0.1 M, adjusted to pH = 2.6 by HCl) together, then add 1.585 g of CTAB and 3050 μL of TEOS. After their complete dissolution, the solution was stirred for 2.5 h at room temperature for hydrolyzation. The assembly of VMSF was conducted under a galvanostatic condition (*j* = −0.70 mA/cm^2^) for 10 s after immersing the ITO in the hydrolyzed sol, using an Ag/AgCl (saturated KCl) as reference electrode and a platinum plate (2 cm × 4 cm) as counter electrode. The as-prepared electrode was rapidly removed from the sol and washed with copious deionized water, dried with N_2_ stream, and further treated overnight at 120 °C. The obtained electrodes were denoted SM@VMSF/ITO, which retained surfactant micelles (SM) templates in the nanochannels. The templates can be conveniently removed using 0.1 M HCl-ethanol by stirring for 5 min and the obtained electrode modified with open nanochannels was termed VMSF/ITO.

#### 2.3.2. Characterization of VMSF/ITO Electrode

The morphology of VMSF was characterized with a JEM-2100 TEM with an acceleration voltage of 100 kV. The TEM specimen was prepared by dispersing the VMSF peeled off from the VMSF/ITO in ethanol through ultrasonication, and finally dropped onto the copper grids for investigation. The thickness of VMSF was characterized using a SU8010 SEM with an acceleration voltage of 5 kV. Before observation, a fresh cross section was obtained by breaking up the VMSF/ITO and spraying it with gold.

The integrity and permeability of VMSF were investigated with the CV technique using Fe(CN)_6_^3−^ and Ru(NH_3_)_6_^3+^, two types of standard redox probes with different charges.

#### 2.3.3. Fabrication of Immunosensor towards CA 15-3

For the immunosensor fabrication, the construction of a biorecognition interface is of great significance. To immobilize the antibody on the outer surface of the VMSF/ITO electrode, the bifunctional reagent GPTMS with both epoxy and silyl groups was selected as the crosslinker [34]. Typically, the SM@VMSF/ITO was immersed in GPTMS (2.26 mM in ethanol) for 60 min, then the GPTMS was covalently linked to the outer surface instead of the inner channels of the VMSF through silane reaction. The electrode was then treated with 0.1 M HCl-ethanol to remove the SM templates. The electrode modified with epoxy-functionalized nanochannels was named O-VMSF/ITO. Anti-CA 15-3 antibodies (anti-CA 15-3) were further immobilized by covering the O-VMSF/ITO with 50 μL of anti-CA 15-3 solution (10 μg/mL in PBS) for 90 min at 37 °C via the ring opening reaction between active epoxy groups on the VMSF and amino residues of the anti-CA 15-3. After rinsing with PBS, it was incubated with s BSA solution (1%, *w*/*w*) for 90 min to block the nonspecific binding sites. The immunosensor termed as anti-CA 15-3/O-VMSF/ITO was obtained and was stored at 4 °C for further tests. The procedure of the immunosensor fabrication is illustrated in Scheme 1.

#### 2.3.4. Dual-Mode Tests

Before the ECL and EC tests, the immunosensor was incubated with different concentrations of CA 15-3 (50 μL in PBS) for 45 min at 37 °C and was noted as CA 15-3/anti-CA 15-3/O-VMSF/ITO, followed by thoroughly washing with PBS. For ECL measurement, the CA 15-3/anti-CA 15-3/O-VMSF/ITO was placed in PBS containing 10 μM Ru(bpy)_3_^2+^ and 3 mM TPA. The ECL process was triggered by a continuous CV procedure with the potential range of 0 to 1.25 V at 0.1 V/s, which was recorded simultaneously along with the ECL signals. The voltage of the photomultiplier tube (PMT) was set at 400 V. For the EC measurement, the CA 15-3/anti-CA 15-3/O-VMSF/ITO was immersed in PBS including 10 μM Ru(bpy)_3_^2+^ and let to stand for 30 min to preconcentration before recording the DPV curves.

Real sample analysis was conducted on human serum from healthy adults using the standard addition method without complex preprocessing. The serum was spiked with a known amount of CA 15-3, followed by 50-times dilution with PBS, and was analyzed by the established methods.

## 3. Results and Discussions

### 3.1. Morphology and Permeability of VMSF/ITO

**Figure 1:**
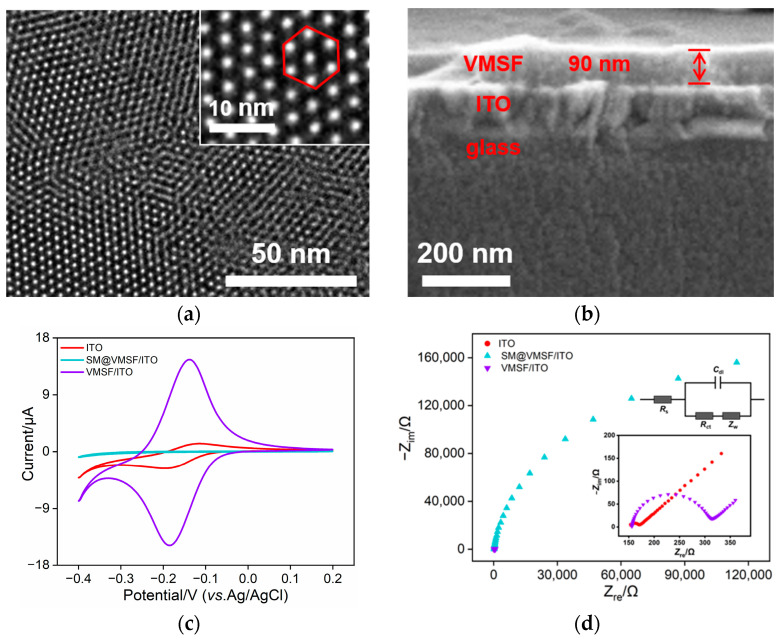
(**a**) Top-view of TEM images and (**b**) SEM image of VMSF. The inset in (**a**) is the corresponding TEM image with high magnification. (**c**) CV plots of ITO, SM@VMSF/ITO, and VMSF/ITO in 0.5 mM Ru(NH_3_)_6_^3+^ and (**d**) EIS plots of ITO, SM@VMSF/ITO, and VMSF/ITO in 0.1 M KCl solution containing 2.5 mM Fe(CN)_6_^3−/4−^. The scan rate in (**c**) was 0.05 V/s. The insets up and down in (**d**) are the equivalent circuit and magnified EIS plots of ITO and VMSF/ITO, respectively.

As expected, the VMSF was assembled on the surface of the ITO electrode due to polycondensation of TEOS under the catalysis of hydroxide ions generated from the electroreduction of protons/water and nitrate ions, where CTAB served as the template, and was proved by TEM and SEM images (Figure 1a,b). VMSF presents a 2D structure of intact film with a thickness of 90 nm and is free of any cracks. A too thin VMSF would lead to an inadequate enrichment of Ru(bpy)_3_^2+^, and would finally fail to achieve a high sensitivity. With the prolongation of electrodeposition time, a much thicker VMSF with more silica aggregates byproducts on its surface would be obtained, which would restrict the diffusion of Ru(bpy)_3_^2+^ species across the film to the underlying electrode surface. According to the many reported works [23,24,28,29,30], a procedure of electrodeposition for 10 s at *j* = −0.70 mA/cm^2^ was selected here to obtain a VMSF with an appropriate thickness. This not only achieves an effective electrostatic attraction to Ru(bpy)_3_^2+^, but also guarantees an efficient and rapid response. The film contains numerous nanochannels that are highly ordered and hexagonally packed and whose diameter is 2~3 nm.

Considering that VMSF plays the role of the modification material that is on the electrolyte/electrode interface, it is crucial to determine how VMSF affects the mass transfer. The permeability is investigated by comparing the electrochemical behaviors of Ru(NH_3_)_6_^3+^ and Fe(CN)_6_^3−/4−^ on ITO, SM@VMSF/ITO and VMSF/ITO. As shown in Figure 1c,d, SM@VMSF/ITO exhibited no peak current but only a non-faradic current towards the Ru(NH_3_)_6_^3+^ probe and a rather large charge transfer resistance (*R*_ct_) represented by the equivalent diameter of Nyquist curve obtained using the EIS technique, a powerful tool to study interfacial property [44]. This is due to the hydrophobic SM templates inside the nanochannels that block the mass transfer of charged probes (Ru(NH_3_)_6_^3+^ and Fe(CN)_6_^3−/4−^) and finally hinder the electron exchange. The nanochannels open after the removal of SM, which facilitates the diffusion of particles, and thus VMSF/ITO shows a pair of well-defined redox peaks to Ru(NH_3_)_6_^3+^ and a much smaller *R*_ct_. In addition, VMSF/ITO exhibits a relatively higher peak current towards Ru(NH_3_)_6_^3+^ than that of ITO, which derives from the deprotonation of abundant silanol with a p*K*_a_ of ~2 in the nanochannel walls that can create a strong electrostatic attraction to positively charged species [25].

The excellent capability of VMSF to enrich cations was further verified by Ru(bpy)_3_^2+^, a bifunctional probe with outstanding ECL and EC activities. The ECL process was triggered by a continuous CV procedure; the mechanism is as follows:

Ru(bpy)_3_^2+^ − e^−^ → Ru(bpy)_3_^3+^

TPA − e^−^ → TPA^·+^

TPA^·+^ − H^+^ → TPA

TPA^·^ + Ru(bpy)_3_^3+^ → Ru(bpy)_3_^2+^*

Ru(bpy)_3_^2+^* → Ru(bpy)_3_^2+^ + *hν* (λ_max_ ≈ 620 nm)

**Figure 2:**
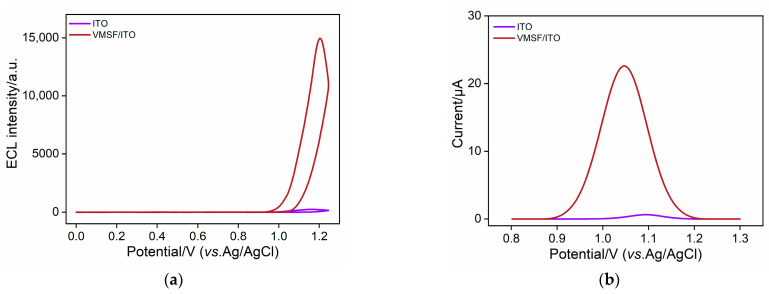
(**a**) ECL curves of ITO and VMSF/ITO in 10 μM Ru(bpy)_3_^2+^ and 3 mM TPA; (**b**) DPV curves of ITO and VMSF/ITO in 10 μM Ru(bpy)_3_^2+^.

During the positive scanning of potential, TPA and Ru(bpy)_3_^2+^ were electrooxidized to TPA^·^ radical and Ru(bpy)_3_^3+^. The redox reaction between TPA^·^ radical and Ru(bpy)_3_^3+^ led to the generation of excited Ru(bpy)_3_^2+^* that emitted light (λ_max_ ≈ 620 nm) when turning to the ground state, which was recorded by the photomultiplier. As shown in Figure 2, the ECL and DPV signals produced by 10 μM Ru(bpy)_3_^2+^ on ITO were very weak while those obtained on VMSF/ITO were remarkable enhanced thanks to the electrostatic preconcentration of the nanochannel layer in the positively charged probe. VMSF/ITO tends to provide an adequate basic signal and exhibits enormous potential in the subsequent immunosensor fabrication.

### 3.2. Characterization of the Immunosensor Construction

**Figure 3:**
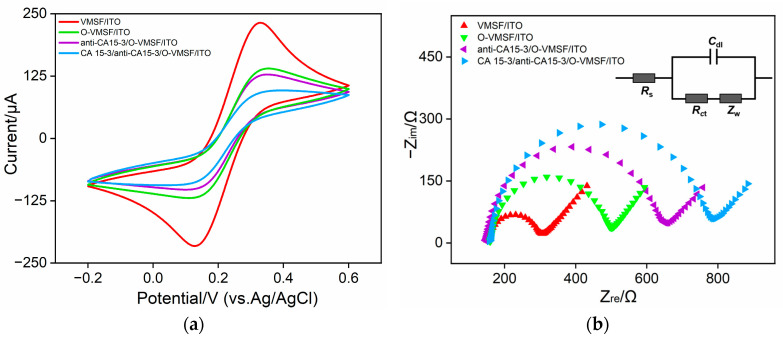
CV (**a**) and EIS (**b**) plots of VMSF/ITO, O-VMSF/ITO, anti-CA 15-3/O-VMSF/ITO, and CA 15-3/anti-CA 15-3/O-VMSF/ITO in a 0.1 M KCl solution containing 2.5 mM Fe(CN)_6_^3−/4−^. The scan rate in (**a**) was 0.05 V/s.

The CV and EIS techniques were employed to monitor the interface features during the bioelectrode’s stepwise assembly. As shown in Figure 3a, VMSF/ITO showed two well-defined redox peaks, which were decreased after the modification of GPTMS, indicating the presence of epoxy group entities. The peak currents were further reduced with the immobilization of antibodies and the capture of CA 15-3 antigens, suggesting that an insulating protein layer was formed on the electrode surface, creating an obstacle to both mass and electron transfer. Figure 3b shows the EIS results fitted according to the Randles equivalent circuit. As shown by the inset, the Randles equivalent circuit contained a solution resistance (*R*_s_), a double-layer capacitance (*C*_dl_), Warburg impedance (*Z*_w_), and an apparent charge transfer resistance (*R*_ct_) that is equal to the equivalent diameter of the semicircle in the high-frequency region [45]. The *R*_ct_ of VMSF/ITO was the smallest (141 Ω) and increased after the crosslinking of GPTMS (329 Ω). The incubation with the antibodies caused an obstruction to the electron transfer of the redox probe, increasing the *R*_ct_ of anti-CA 15-3/O-VMSF/ITO significantly (493 Ω). The bio-recognition and combination of CA 15-3 further aggravated the blocking effect and CA 15-3/anti-CA 15-3/O-VMSF/ITO had the largest *R*_ct_ (607 Ω). The results above indicate the successful fabrication of the immunosensor.

### 3.3. Dual-Mode Tests of CA 15-3

**Figure 4:**
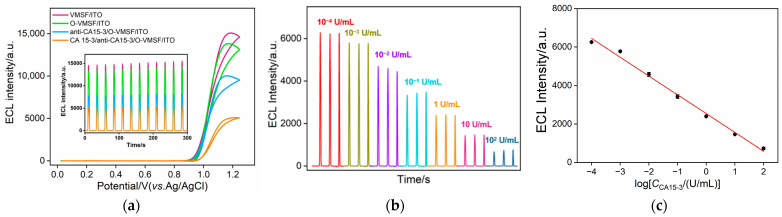
(**a**) IECL-potential curves of VMSF/ITO, O-VMSF/ITO, anti-CA 15-3/O-VMSF/ITO, and CA 15-3/anti-CA 15-3/O-VMSF/ITO in 10 μM Ru(bpy)32+. The inset in (**a**) shows the corresponding IECL-time curves. (**b**) ECL responses of the immunosensor to various concentrations of CA 15-3 from 10^−4^ U/mL, 10^−3^ U/mL, 10^−2^ U/mL, 10^−1^ U/mL, 1 U/mL, 10 U/mL to 100 U/mL. (**c**) The corresponding calibration curve. The error bars represent the standard deviation of three measurements under the same condition.

The specific recognition and combination between the anti-CA 15-3 antibody and the CA 15-3 antigen formed an antibody-antigen immunocomplex. In analogy with the aforementioned CV and EIS results, Figure 4a shows decreased ECL responses with the step-by-step assembling of the bioelectrode, confirming the feasibility of the ECL analysis. The inset suggested an outstanding stability of the biosensor under continuous CV scanning with a relative standard deviation (RSD) of less than 2%. The *I*_ECL_-time curves recorded on bioelectrodes incubated with a series of different amounts of CA 15-3 are shown in Figure 4b. With the increase in CA 15-3, more immunocomplex was formed, which perturbed the mass transfer of Ru(bpy)_3_^2+^ and restrained the electron transfer at the electrode. A linear relationship for CA 15-3 was obtained from 0.1 mU/mL to 100 U/mL using the equation *I*_ECL_ = −997.0 log*C*_CA15-3_ + 2537 (R^2^ = 0.991). LOD is defined by the International Union of Pure and Applied Chemistry (IUPAC) as the concentration (*c*_L_) that can be detected with a reasonable certainty for a given analytical procedure. It can be derived from the smallest measure (*x*_L_), which is calculated with the equation *x*_L_ = *x*_b1_ ± *ks*_b1_, where *x*_b1_ is the mean of the blank measures, *s*_b1_ is the standard deviation of the blank measures, and *k* is a numerical factor commonly recommended as 3. Here, + and − are applied to signal-on and signal-off sensors, respectively [46]. Thus, the LOD was calculated to be 9 μU/mL according to the calibration curve equation. Compared with the other reported methods listed in Table 1, this proposed ECL immunosensor exhibits a relatively wider linear range and an exceedingly lower LOD for CA 15-3 determination without elaborate and complex labor during the construction process. Diverse sophisticated nanomaterials and sandwich strategies are widely used in other related works listed in Table 1. They require a complex preparation process, several steps of incubation, and consume more high-cost biological reagents. Our proposed sensor is simple in preparation, does not need multiple incubations for detection, and is much more economic.

**Figure 5:**
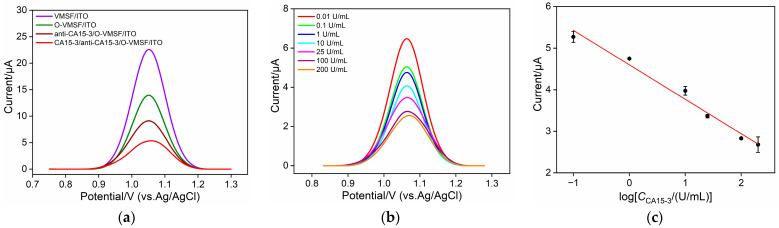
(**a**) DPV curves of VMSF/ITO, O-VMSF/ITO, anti-CA 15-3/O-VMSF/ITO, and CA 15-3/anti-CA 15-3/O-VMSF/ITO in 10 μM Ru(bpy)_3_^2+^. (**b**) DPV response of anti-CA 15-3/O-VMSF/ITO in the presence of different concentrations of CA 15-3. (**c**) The corresponding calibration curve. The error bars represent the standard deviation of three measurements under the same condition.

In addition, considering the excellent electroactivity of Ru(bpy)_3_^2+^, CA 15-3 was quantified using the DPV method. Similar to the ECL mode, the feasibility of the EC mode was verified by the gradually reduced DPV response during the bioelectrode fabrication as shown in Figure 5a. Upon the increase in CA 15-3, a group of peak currents which decreased linearly with the logarithm of the antigen concentration from 10 mU/mL to 200 U/mL were obtained (Figure 5b), and the calibration curve was represented in Figure 5c using the equation *I* = −0.82 log*C*_CA15-3_ + 4.60 (R^2^ = 0.992). Similar to the ECL mode, the LOD was calculated to be 5.4 mU/mL (S/N = 3). Although the EC method was inferior in LOD compared with the ECL mode, a higher detection upper limit was achieved. Although the LOD of our proposal is not as low as 10^−5^ U/mL for the sandwich strategy using Ab_2_/HRP-MBs and Ab_1_/AuE, our immunosensor is free of complex labeling operations or several steps of incubation. Dual-mode tests that combine the merits of ECL and EC strategies succeed to broaden the working linear range while achieving an ultrasensitive LOD. The total cost of a single fabricated immunosensor is approximately 0.366 USD, which is much lower in cost compared to ELISA which costs around 1.31 USD for a single sample test. Furthermore, the materials involved here are all commercially available, indicating its potential for industrialization, as well as the possibility of application in microfluidic Point-of-Care devices [47,48]. However, the immunosensor is susceptible to matrix effects, and a dilution is required before testing bio-samples to guarantee the accuracy in clinical application. In addition, an alkali condition may lead to the hydrolysis of VMSF as the proposed immunosensor is applicative in neutral medium to keep its mechanical stability. The current clinical determination of CA 15-3 is mainly based on a chemiluminescence immunoassay. This method not only involves labeling technology, but also requires two antibodies, as well as several steps of incubation and washing, which is high-cost and time-consuming. Our proposed method involves only one step of incubation, which meets the needs of rapid clinical determination of CA 15-3 at low cost.

**Table 1:** Comparison of various methods for the determination of CA 15-3.

Electrode	Mode	Classification	Linear Range (U/mL)	LOD (μU/mL)	Ref.
anti-CA 15-3/NH_2_-SiO_2_-PTCA/CeO_2_/Pt/rGO/GCE	ECL	Label-free	0.012–120	1348	[49]
anti-CA 15-3/Ru(bpy)_3_^2+^@UiO-66-NH_2_/GCE	ECL	Label-free	5 × 10^−4^–500	17.705	[50]
Ab_2_/AuNPs/CQDs-PEI-GO and Ab_1_/AgNPs-PDA/GCE	ECL	Labeled	5 × 10^−3^–500	1.7 × 10^3^	[51]
anti-CA 15-3/CS/PtNi NCs-*L*-Cys-luminol/GCE	ECL	Label-free	5 × 10^−4^–500	167	[52]
Ab_2_/CdTe@CdS/PAMAM and Ab_2_/Fe_3_O_4_@SiO_2_, ITO	ECL	Labeled	10^−4^–100	10	[53]
Ab_2_/HRP-MBs and Ab_1_/AuE	EC	Label-free	1.5 × 10^−5^–50	15	[54]
anti-CA 15-3/Ag/TiO_2_/rGO/GCE	EC	Label-free	0.1–300	7 × 10^4^	[55]
anti-CA 15-3/DAP-AuNPs/P3ABA/2D-MoSe_2_/GO/SPCE	EC	Label-free	0.14–500	1.4 × 10^5^	[56]
anti-CA 15-3/O-VMSF/ITO	ECL	Label-free	10^−4^–100	9	This work
	EC		10^−2^–100	5.4 × 10^3^	

PTCA: 3,4,9,10-perylenetetracarboxylic acid; rGO: reduced graphene oxide; GCE: glassy carbon electrode; Ab: antibody; AuNPs: gold nanoparticles; CQDs: carbon quantum dots; PEI: polyethylenimine; GO: graphene oxide; AgNPs: silver nanoparticles; PDA: polydopamine; PtNi NCs: platinum nickel nanocubes; *L*-Cys: *L*-cysteine; PAMAM: polyamidoamine; HRP: horseradish peroxidase; MBs: magnetic beads; AuE: gold electrode; DAP: deposited redox dye; P3ABA: poly(3-aminobenzylamine); and SPCE: screen-printed carbon electrode.

### 3.4. Specificity, Selectivity and Stability of the Immunosensor

**Figure 6:**
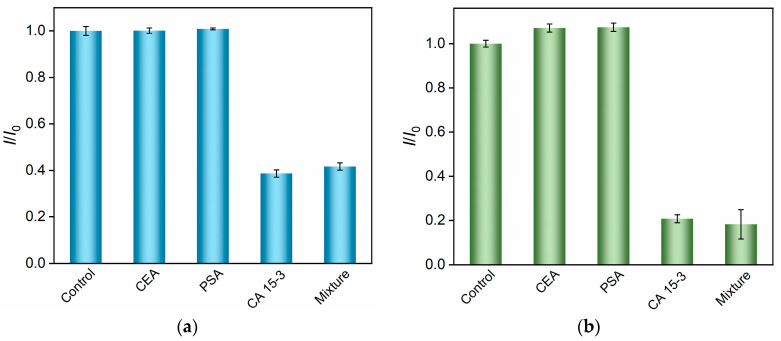
Relative ratio of ECL intensity (**a**) and DPV current (**b**) before (*I*_0_) and after (*I*) incubation with buffer (Control), CEA (500 ng/mL), PSA (500 ng/mL), CA 15-3 (25 U/mL), or a protein mixture (25 U/mL CA 15-3 + 500 ng/mL other proteins). The error bars represent the standard deviation of three measurements under the same condition.

The proposed immunosensor was expected to be highly specific and selective for CA 15-3 detection due to the antigen-antibody recognition. Therefore, the bioelectrode was tested with different possible clinical cancer biomarkers such as the carcinoembryonic antigen (CEA) and the prostate specific antigen (PSA) (the concentrations of interfering proteins are both 500 ng/mL). As illustrated in Figure 6, the signal fluctuation caused by individual interfering proteins was less than 7%; an evident decrease in signal was observed when incubated with CA 15-3 or the mixture of all proteins. These results indicated the high specificity and outstanding selectivity of the fabricated immunosensor. The stability of the anti-CA 15-3/O-VMSF/ITO was evaluated by comparing the signal after several days of storage at 4 °C with the initial signal on the first day. Anti-CA 15-3/O-VMSF/ITO retained more than 95% of the initial signal after a 15-day storage at 4 °C, suggesting a satisfactory stability.

### 3.5. Real Sample Analysis

To validate the practical applicability of the established method, the biosensor was used to determine the CA 15-3 spiked in healthy human serum samples. A series of known amount of CA 15-3 was added to the samples and then they were diluted with PBS 50 times. After the incubation with anti-CA 15-3/O-VMSF/ITO, the CA 15-3/anti-CA 15-3/O-VMSF/ITO was obtained for ECL and EC testing. The concentration of CA 15-3 could be calculated by substituting the obtained ECL and EC response into the original linear equation. The results are listed in Table 2. Satisfactory recoveries ranging from 95.4% to 104.0% and an RSD below 2.9% were obtained using the ECL-EC dual-mode tests, which implied the potential for clinical application.

## 4. Conclusions

In summary, an ECL-EC dual-mode immunosensor with a simple fabrication strategy was designed for CA 15-3 determination with an efficient biosensing performance. The VMSF equipped on the electrode surface displays a strong electrostatic attraction to the positively charged bifunctional probes, and provides a large surface area with good biocompatibility for the biorecognition of elements binding with the aid of the crosslinker GPTMS. Integrating the merits of both the sensitivity of electrochemiluminescence and the efficiency of electrochemistry, the immunosensor demonstrated a satisfactory performance with a linear working range of more than 7 orders of magnitude and a LOD of 9 μU/mL. Furthermore, VMSF exhibits good resistance to foulants thanks to the ultrasmall diameter of nanochannels. Real sample analysis in human serum was performed, expecting to provide a new method for clinical CA 15-3 diagnosis.

## Figures and Tables

**Table 2:** Detection of CA 15-3 in human serum.

Mode	Sample *	Added (U/mL)	Found (U/mL)	RSD (%)	Recovery (%)
ECL	1	1.00	0.980	2.1	98.0
		5.00	4.80	1.5	96.0
		10.0	10.1	2.0	101.0
	2	1.00	1.04	2.7	104.0
		5.00	5.12	2.9	102.4
		10.0	9.86	2.0	98.6
EC	1	5.00	4.77	1.0	95.4
		50.0	49.5	1.7	99.0
		100.0	99.6	2.1	99.6
	2	5.00	4.86	1.8	97.2
		50.0	51.5	2.0	103.0
		100.0	102.2	2.7	102.2

* Diluted by PBS for 50 times and CA 15-3 was added before dilution.

## Data Availability

The data presented in this study are available on request from the corresponding author.

## References

[1] Bray F., Ferlay J., Soerjomataram I., Siegel R.L., Torre L.A., Jemal A. (2018). Global cancer statistics 2018: GLOBOCAN estimates of incidence and mortality worldwide for 36 cancers in 185 countries. CA-Cancer J. Clin..

[2] DeSantis C., Ma J., Bryan L., Jemal A. (2014). Breast cancer statistics, 2013. CA-Cancer J. Clin..

[3] Jing L., Xie C., Li Q., Yang M., Li S., Li H., Xia F. (2022). Electrochemical biosensors for the analysis of breast cancer biomarkers: From design to application. Anal. Chem..

[4] Rifai N., Gillette M.A., Carr S.A. (2006). Protein biomarker discovery and validation: The long and uncertain path to clinical utility. Nat. Biotechnol..

[5] Carneiro M., Rodrigues L., Moreira F., Goreti F., Sales M. (2022). Paper-based ELISA for fast CA 15-3 detection in point-of-care. Microchem. J..

[6] Marques R., Costa-Rama E., Viswanathan S., Nouws H., Costa-García A., Delerue-Matos C., González-García M. (2018). Voltammetric immunosensor for the simultaneous analysis of the breast cancer biomarkers CA 15-3 and HER2-ECD. Sens. Actuators B Chem..

[7] Hasanzadeh M., Tagi S., Solhi E., Mokhtarzadeh A., Shadjou N., Eftekhari A., Mahboob S. (2018). An innovative immunosensor for ultrasensitive detection of breast cancer specific carbohydrate (CA 15-3) in unprocessed human plasma and MCF-7 breast cancer cell lysates using gold nanospear electrochemically assembled onto thiolated graphene quantum dots. Int. J. Biol. Macromol..

[8] Ambrosi A., Airo F., Merkoçi A. (2010). Enhanced gold nanoparticle based ELISA for a breast cancer biomarker. Anal. Chem..

[9] Huang X., Liu H., Fang W., Lin Y., Tan Y. (2018). Sensitive and selective immunofluorescence assay for CA15-3 detection using fluorescein derivative A10254. Protein Pept. Lett..

[10] Yang Y., Zhang H., Zhang M., Meng Q., Cai L., Zhang Q. (2017). Elevation of serum CEA and CA15-3 levels during antitumor therapy predicts poor therapeutic response in advanced breast cancer patients. Oncol. Lett..

[11] Zhou H., Ma X., Sailjoi A., Zou Y., Lin X., Yan F., Su B., Liu J. (2022). Vertical silica nanochannels supported by nanocarbon composite for simultaneous detection of serotonin and melatonin in biological fluids. Sens. Actuators B Chem..

[12] Xuan L., Liao W., Wang M., Zhou H., Ding Y., Yan F., Liu J., Tang H., Xi F. (2021). Integration of vertically-ordered mesoporous silica-nanochannel film with electro-activated glassy carbon electrode for improved electroanalysis in complex samples. Talanta.

[13] Hu Y., Zhu L., Mei X., Liu J., Yao Z., Li Y. (2021). Dual-mode sensing platform for electrochemiluminescence and colorimetry detection based on a closed bipolar electrode. Anal. Chem..

[14] Mi X., Li H., Tan R., Tu Y. (2020). Dual-modular aptasensor for detection of cardiac troponin I based on mesoporous silica films by electrochemiluminescence/electrochemical impedance spectroscopy. Anal. Chem..

[15] Kuo S., Li H., Wu P., Chen C., Huang Y., Chan Y. (2015). Dual colorimetric and fluorescent sensor based on semiconducting polymer dots for ratiometric detection of lead ions in living cells. Anal. Chem..

[16] Xue J., Zhao Q., Yang L., Ma H., Wu D., Liu L., Ren X., Ju H., Wei Q. (2021). Dual-mode sensing platform guided by intramolecular electrochemiluminescence of a ruthenium complex and cationic N,N-Bis(2-(trimethylammonium iodide)propylene) Perylene-3,4,9,10-tetracarboxydiimide for estradiol assay. Anal. Chem..

[17] Gong J., Zhang T., Chen P., Yan F., Liu J. (2022). Bipolar silica nanochannel array for dual-mode electrochemiluminescence and electrochemical immunosensing platform. Sens. Actuators B Chem..

[18] Ma K., Zheng Y., An L., Liu J. (2022). Ultrasensitive immunosensor for prostate-specific antigen based on enhanced electrochemiluminescence by vertically ordered mesoporous silica-nanochannel film. Front. Chem..

[19] Zhao T., Elzatahry A., Li X., Zhao D. (2019). Single-micelle-directed synthesis of mesoporous materials. Nat. Rev. Mater..

[20] Liu X., Chen Z., Wang T., Jiang X., Qu X., Duan W., Xi F., He Z., Wu J. (2022). Tissue imprinting on 2D nanoflakes-capped silicon nanowires for lipidomic mass spectrometry imaging and cancer diagnosis. ACS Nano.

[21] Cui Y., Duan W., Jin Y., Wo F., Xi F., Wu J. (2020). Ratiometric fluorescent nanohybrid for noninvasive and visual monitoring of sweat glucose. ACS Sens..

[22] Zhou L., Hou H., Wei H., Yao L., Sun L., Yu P., Su B., Mao L. (2019). In vivo monitoring of oxygen in rat brain by carbon fiber microelectrode modified with antifouling nanoporous membrane. Anal. Chem..

[23] Deng X., Lin X., Zhou H., Liu J., Tang H. (2023). Equipment of vertically-ordered mesoporous silica film on electrochemically pretreated three-dimensional graphene electrodes for sensitive detection of methidazine in urine. Nanomaterials.

[24] Yang L., Zhang T., Zhou H., Yan F., Liu Y. (2022). Silica nanochannels boosting Ru(bpy)_3_^2+^-mediated electrochemical sensor for the detection of guanine in beer and pharmaceutical samples. Front. Nutrit..

[25] Mathieu E., Alida Q., David G., Lionel N., Clement S., Alain W. (2007). Molecular transport into mesostructured silica thin films: Electrochemical monitoring and comparison between *p*6*m*, P6_3_/*mmc*, and *Pm*3*n* structures. Chem. Mater..

[26] Nasir T., Herzog G., Hébrant M., Despas C., Liu L., Walcarius A. (2018). Mesoporous silica thin films for improved electrochemical detection of paraquat. ACS Sens..

[27] Zhu X., Xuan L., Gong J., Liu J., Wang X., Xi F., Chen J. (2022). Three-dimensional macroscopic graphene supported vertically-ordered mesoporous silica-nanochannel film for direct and ultrasensitive detection of uric acid in serum. Talanta.

[28] Zhou H., Ding Y., Su R., Lu D., Tang H., Xi F. (2022). Silica nanochannel array film supported by ß-cyclodextrin-functionalized graphene modified gold film electrode for sensitive and direct electroanalysis of acetaminophen. Front. Chem..

[29] Zhang M., Zou Y., Zhou X., Yan F., Ding Z. (2022). Vertically-ordered mesoporous silica films for electrochemical detection of Hg(II) ion in pharmaceuticals and soil samples. Front. Chem..

[30] Yan F., Luo T., Jin Q., Zhou H., Sailjoi A., Dong G., Liu J., Tang W. (2021). Tailoring molecular permeability of vertically-ordered mesoporous silica-nanochannel films on graphene for selectively enhanced determination of dihydroxybenzene isomers in environmental water samples. J. Hazard. Mater..

[31] Zhou C., Yan J., Chen B., Li P., Dong X., Xi F., Liu J. (2016). Synthesis and application of ternary photocatalyst with a gradient band structure from two-dimensional nanosheets as precursors. RSC Adv..

[32] Gong J., Zhang T., Luo T., Luo X., Yan F., Tang W., Liu J. (2022). Bipolar silica nanochannel array confined electrochemiluminescence for ultrasensitive detection of SARS-CoV-2 antibody. Biosens. Bioelectron..

[33] Yan F., Chen J., Jin Q., Zhou H., Sailjoi A., Liu J., Tang W. (2020). Fast one-step fabrication of a vertically-ordered mesoporous silica-nanochannel film on graphene for direct and sensitive detection of doxorubicin in human whole blood. J. Mater. Chem. C.

[34] Saadaoui M., Fernández I., Sánchez A., Díez P., Campuzano S., Raouafi N., Pingarrón J., Villalonga R. (2015). Mesoporous silica thin film mechanized with a DNAzyme-based molecular switch for electrochemical biosensing. Electrochem. Commun..

[35] Serrano M.B., Despas C., Herzog G., Walcarius A. (2015). Mesoporous silica thin films for molecular sieving and electrode surface protection against biofouling. Electrochem. Commun..

[36] Lv N., Qiu X., Han Q., Xi F., Wang Y., Chen J. (2022). Anti-biofouling electrochemical sensor based on the binary nanocomposite of silica nanochannel array and graphene for doxorubicin detection in human serum and urine samples. Molecules.

[37] Zheng W., Su R., Yu G., Liu L., Yan F. (2022). Highly sensitive electrochemical detection of paraquat in environmental water samples using a vertically ordered mesoporous silica film and a nanocarbon composite. Nanomaterials.

[38] Lu L., Zhou L., Chen J., Yan F., Liu J., Dong X., Xi F., Chen P. (2018). Nanochannel-confined graphene quantum dots for ultrasensitive electrochemical analysis of complex samples. ACS Nano.

[39] Zou Y., Zhou X., Xie L., Tang H., Yan F. (2022). Vertically-ordered mesoporous silica films grown on boron nitride-graphene composite modified electrodes for rapid and sensitive detection of carbendazim in real samples. Front. Chem..

[40] Zhou Z., Guo W., Xu L., Yang Q., Su B. (2015). Two orders-of-magnitude enhancement in the electrochemiluminescence of Ru(bpy)_3_^2+^ by vertically ordered silica mesochannels. Anal. Chim. Acta.

[41] Yan F., Ma X., Jin Q., Tong Y., Tang H., Lin X., Liu J. (2020). Phenylboronic acid-functionalized vertically ordered mesoporous silica films for selective electrochemical determination of fluoride ion in tap water. Microchim. Acta.

[42] Yan L., Xu S., Xi F. (2022). Disposal immunosensor for sensitive electrochemical detection of prostate-specific antigen based on amino-rich nanochannels array-modified patterned indium tin oxide electrode. Nanomaterials.

[43] Huang L., Su R., Xi F. (2023). Sensitive detection of noradrenaline in human whole blood based on Au nanoparticles embedded vertically-ordered silica nanochannels modified pre-activated glassy carbon electrodes. Front. Chem..

[44] Fang D., Zhang S., Dai H., Lin Y. (2019). An ultrasensitive ratiometric electrochemiluminescence immunosensor combining photothermal amplification for ovarian cancer marker detection. Biosens. Bioelectron..

[45] Aydin E., Aydin M., Sezginturk M. (2021). New impedimetric sandwich immunosensor for ultrasensitive and highly specific detection of spike receptor binding domain protein of SARS-CoV-2. ACS Biomater. Sci. Eng..

[46] Chen L., Li Y., Miao L., Pang X., Li T., Qian Y., Li H. (2021). “Lighting-up” curcumin nanoparticles triggered by pH for developing improved enzyme-linked immunosorbent assay. Biosens. Bioelectron..

[47] Yin B., Wan X., Yang M., Qian C., Muhtasim Fuad Sohan A.S.M. (2022). Wave-shaped microfluidic chip assisted point-of-care testing for accurate and rapid diagnosis of infections. Mil. Med. Res..

[48] Yin B., Qian C., Wan X., Muhtasim Fuad Sohan A.S.M., Lin X. (2022). Tape integrated self-designed microfluidic chip for point-of-care immunoassays simultaneous detection of disease biomarkers with tunable detection range. Biosens. Bioelectron..

[49] Lin Z., Zheng S., Xie J., Zhou R., Chen Y., Gao W. (2023). A sensitive electrochemiluminescence immunosensor for the detection of CA15-3 based on CeO_2_/Pt/rGO as a novel co-reaction accelerator. Talanta.

[50] Xiong X., Zhang Y., Wang Y., Sha H., Jia N. (2019). One-step electrochemiluminescence immunoassay for breast cancer biomarker CA 15-3 based on Ru(bpy)_3_^2+^-coated UiO-66-NH_2_ metal-organic framework. Sens. Actuators B Chem..

[51] Qin D., Jiang X., Mo G., Feng J., Yu C., Deng B. (2019). A novel carbon quantum dots signal amplification strategy coupled with sandwich electrochemiluminescence immunosensor for the detection of CA15-3 in human serum. ACS Sens..

[52] Wang Y., Sha H., Ke H., Jia N. (2018). An ultrasensitive electrochemiluminescence immunosensor based on platinum nickel nanocubes-*L*-cysteine-luminol nanocomposite. Talanta.

[53] Babamiri B., Hallaj R., Salimi A. (2018). Ultrasensitive electrochemiluminescence immunoassay for simultaneous determination of CA125 and CA15-3 tumor markers based on PAMAM-sulfanilic acid-Ru(bpy)_3_^2+^ and PAMAM-CdTe@CdS nanocomposite. Biosens. Bioelectron..

[54] Akbari Nakhjavani S., Khalilzadeh B., Samadi Pakchin P., Saber R., Ghahremani M., Omidi Y. (2018). A highly sensitive and reliable detection of CA15-3 in patient plasma with electrochemical biosensor labeled with magnetic beads. Biosens. Bioelectron..

[55] Shawky A., El-Tohamy M. (2021). Signal amplification strategy of label-free ultrasenstive electrochemical immunosensor based ternary Ag/TiO_2_/rGO nanocomposites for detecting breast cancer biomarker CA 15-3. Mater. Chem. Phys..

[56] Pothipor C., Bamrungsap S., Jakmunee J., Ounnunkad K. (2022). A gold nanoparticle-dye/poly(3-aminobenzylamine)/two dimensional MoSe_2_/graphene oxide electrode towards label-free electrochemical biosensor for simultaneous dual-mode detection of cancer antigen 15-3 and microRNA-21. Colloids Surf. B Biointerfaces.

